# Dysregulation of fragile X mental retardation protein and metabotropic glutamate receptor 5 in superior frontal cortex of individuals with autism: a postmortem brain study

**DOI:** 10.1186/2040-2392-2-6

**Published:** 2011-05-06

**Authors:** S Hossein Fatemi, Timothy D Folsom

**Affiliations:** 1 Division of Neuroscience Research, Department of Psychiatry, University of Minnesota Medical School, 420 Delaware Street SE, MMC 392, Minneapolis, MN 55455, USA; 2 Department of Pharmacology, University of Minnesota Medical School, 420 Delaware Street SE, MMC 392, Minneapolis, MN 55455, USA; 3 Department of Neuroscience, University of Minnesota Medical School, 420 Delaware Street SE, MMC 392, Minneapolis, MN 55455, USA

## Abstract

**Background:**

Fragile X syndrome is caused by loss of function of the fragile X mental retardation 1 (*FMR1*) gene and shares multiple phenotypes with autism. We have previously found reduced expression of the protein product of *FMR1 *(FMRP) in vermis of adults with autism.

**Methods:**

In the current study, we have investigated levels of FMRP in the superior frontal cortex of people with autism and matched controls using Western blot analysis. Because FMRP regulates the translation of multiple genes, we also measured protein levels for downstream molecules metabotropic glutamate receptor 5 (mGluR5) and γ-aminobutyric acid (GABA) A receptor β3 (GABRβ3), as well as glial fibrillary acidic protein (GFAP).

**Results:**

We observed significantly reduced levels of protein for FMRP in adults with autism, significantly increased levels of protein for mGluR5 in children with autism and significantly increased levels of GFAP in adults and children with autism. We found no change in expression of GABRβ3. Our results for FMRP, mGluR5 and GFAP confirm our previous work in the cerebellar vermis of people with autism.

**Conclusion:**

These changes may be responsible for cognitive deficits and seizure disorder in people with autism.

## Background

Autism and fragile X syndrome (FXS) are two disorders that share several commonalities, including reduced cerebellar volume, altered dendritic spine morphology [1-4], presence of seizures, mental retardation and social anxiety [2-5], as well as other behavioral abnormalities [5,6]. People with both FXS and autism have been shown to have lower IQ scores [7], lower adaptive skills [7], lower expressive language skills [8] and greater autonomic dysfunction and hyperarousal [9] than people with FXS alone. The rate of FXS in people with autism varies from 2% to 8% [10,11]. The prevalence of autism in people with FXS has been estimated to be anywhere from 25% to 47% [5,12,13].

All diagnoses of FXS require loss of function mutation of the fragile X mental retardation 1 (*FMR1*) gene [14]. The product of the *FMR1 *gene, fragile X mental retardation protein (FMRP), has roles in multiple intranuclear and posttranscriptional events [15,16] and has been shown to bind to up to 4% of the mRNA expressed in the brain [17]. FMRP is thought to be involved in multiple developmental events, including normal differentiation, migration of subpopulations of neuronal cells and the development of cortical circuits [18]. FMRP may also have roles in synaptic pruning [19], and the results of multiple studies have supported a role for FMRP in synaptic plasticity [17,20]. All of these functions are relevant to the pathology of autism. Any abnormality in FMRP function could affect multiple genes and pathways [21]. For example, reduced FMRP expression has been shown to negatively affect the expression of γ-aminobutyric acid A (GABA_A_) receptors in FXS animal models [22-24], and work done at our laboratory has shown significant reductions in protein for both FMRP and the GABA_A_, β3 subunit (GABRβ3) in vermis of adults with autism [25].

In vermis of people with autism, our laboratory has recently demonstrated the following: (1) reduction in levels of FMRP and GABRβ3 in adults with autism, (2) increased expression of metabotropic glutamate receptor 5 (mGluR5) in children with autism and (3) increased glial fibrillary acidic protein (GFAP) in children and adults with autism [25]. In the current study, we have expanded our research to measure protein levels for the same molecules in the superior frontal cortex (Brodmann's area 9 (BA9)). We hypothesized that FMRP would be reduced, potentially explaining deficits that are common in autism spectrum disorder and FXS.

## Methods

### Tissue preparation

All experimental procedures were approved by the Institutional Review Board of the University of Minnesota School of Medicine. Postmortem blocks of superior frontal cortex (that is, BA9) were obtained from the National Institute of Child Health & Human Development Brain and Tissue Bank for Developmental Disorders, University of Maryland, Baltimore, MD, USA; the Harvard Brain Tissue Resource Center, which is supported in part by Public Health Service grant R24 MH068855; the Brain Endowment Bank, which is funded in part by the National Parkinson Foundation, Inc., Miami, FL, USA; and the Autism Tissue Program. The tissue samples (Table 1) were prepared as described previously [25-28]. Sample B6184 was tested for FXS, and the test results were negative. There are no records to indicate that any of the other individuals received genetic testing for, or were diagnosed with, FXS, duplication at chromosome 15q or any other relevant genetic abnormalities that could contribute to the pathology of autism.

**Table 1:** Demographic data for people with autism and healthy controls^a^

Case	Dx	Sex	Age	PMI, hours	Ethnicity	Medication History	Cause of Death	Seizure	MR
4670	Control	M	4	17	Caucasian	None	*Commotio Cordis*	No	No
1674	Control	M	8	36	Caucasian	None	Drowning	No	No
4787	Control	M	12	15	African American	Montelukast, Albuteral, Prednisone, Loratadine	Asthma	No	No
1823	Control	M	15	18	Caucasian	None	MVA	No	No
6396	Control	M	18	19.83	Unknown	None	Unknown	No	No
1846	Control	F	20	9	Caucasian	None	MVA	No	No
6316	Control	F	32	28.92	Unknown	None	Unknown	No	No
1169	Control	M	33	27	African American	Metoclopramide, Loratadine	Dilated Cardiomyopathy (morbid obesity)	No	No
1376	Control	M	37	12	African American	None	ASCVD	No	No
6420	Control	M	41	30.4	Unknown	None	Heart Attack	No	No
7002	Autism	F	5	32.73	Asian	None	Drowning	No	No
5569	Autism	M	5	39	Caucasian	None	Drowning	No	No
1174	Autism	F	7	14	Caucasian	None	Seizure disorder	Yes	No
5666	Autism	M	8	22.16	Caucasian	None	Cancer	Yes	No
4231	Autism	M	8	12	African American	Olanzapine, Galantamine	Drowning	No	Yes
4925	Autism	M	9	27	Caucasian	Clonidine, Sodium valproate, Phenytoin, Lamotrigine, Methylphenidate, Carbamazepine	Seizure disorder	Yes	No
4899	Autism	M	14	9	Caucasian	None	Drowning	No	No
6184	Autism	F	18	6.75	Caucasian	None	Seizure disorder	Yes	No
5144	Autism	M	20	23.66	Caucasian	Erythromycin gel, Minocycline	MVA	No	Yes
1638	Autism	F	20	50	Caucasian	None	Seizure disorder	Yes	Yes
6337	Autism	M	22	25	Caucasian	Aripiprazole, Lamotrigine, Zonisamide	Aspiration	Yes	No
6994	Autism	M	29	43.25	Caucasian	Fexofenadine, Ziprasidone HCl, Carbamazepine	Seizure (suspected)	Yes	No
6640	Autism	F	29	17.83	Caucasian	Fluvoxamine	Seizure disorder	Yes	No
6677	Autism	M	30	16.06	Caucasian	None	Heart Failure (congestive)	No	No
5173	Autism	M	30	20.33	Caucasian	Cisapride, Clorazepate, Sodium valproate, Phenytoin, Folic Acid, Primidone, Phenobarbital, Omeprazole, Cisapride, Metoclopramide	Gastrointestinal bleeding	Yes	No
5027	Autism	M	37	26	African American	None	Obstruction of bowel due to adhesion	No	No
6401	Autism	M	39	13.95	Caucasian	None	Cardiac Tamonade	No	No
5562	Autism	M	39	22.75	Caucasian	None	Sudden unexpected death	Yes	No
6469	Autism	F	49	16.33	Caucasian	Wafarin, Venlafaxine, Erythromycin, Lansoprazole, Risperidone, Metformin, Gabapentin, Propranolol, Levothyroxine	Pulmonary Arrest	No	Yes
4498	Autism	M	56	19.48	Caucasian	Benztropine mesylate, Haloperidol, Lithium, Chlorpromazine, Alprazolam	Anoxic encephalopathy	Yes	No
									

**Children**	**Control**	**Autistic**	**Change**	***P *** **value**	**Cohen's** ***d***				

Age ± SD (years)	11.4 ± 5.55	9.25 ± 4.53	↓18.9%	ns	--				
PMI ± SD (years)	21.2 ± 8.47	18.6 ± 9.47	↓12.3%	ns	--				
Gender	5M	5M:3F	--	--	--				

**Adults**	**Control**	**Autistic**	**Change**	***P *** **value**	**Cohen's** ***d***				

Age ± SD (years)	32.6 ± 7.89	33.3 ± 11.2	↑2.1%	ns	--				
PMI ± SD (years)	21.5 ± 10.1	24.6 ± 11.1	↑14.4%	ns	--				
Gender	3M:2F	9M:3F	--	--	--				

^a^Dx, diagnosis; PMI, postmortem interval; MVA, motor vehicle accident; MR, mental retardation; ns, not significant.

### Sodium dodecyl sulfate polyacrylamide gel electrophoresis and Western blot analysis

Tissue samples from BA9 (adults: *n *= 5 controls, *n *= 8 autistic; children: *n *= 5 controls, *n *= 12 autistic) were prepared and subjected to sodium dodecyl sulfate polyacrylamide gel electrophoresis (SDS-PAGE) and Western blot analysis as described previously [25-28]. For FMRP and mGluR5, 6% resolving gels were used. For GABRβ3, GFAP and neuron-specific enolase (NSE), 10% resolving gels were used. Following SDS-PAGE, proteins were transferred onto nitrocellulose membranes for 2 hours at 300 mA. Western blots were blocked for 1 hour at room temperature (RT) followed by overnight incubation in primary antibodies at 4°C. The primary antibodies used were anti-FMRP (1:5,000; Millipore, Billerica, MA, USA) anti-mGluR5 (1:300; Abcam Inc., Cambridge, MA, USA), anti-GABRβ3 (1:1,000; Novus Biologicals, Littleton, CO, USA), anti-GFAP (1:2,000; Sigma-Aldrich, St. Louis, MO, USA), anti-NSE (1:2,000; Abcam Inc.) and anti-β-actin (1:5,000; Sigma-Aldrich). Blots were incubated in primary antibodies overnight at 4°C. Following a 30-minute wash in phosphate-buffered saline supplemented with 0.003% Tween 20 (PBST), blots were incubated in secondary antibodies for 60 minutes at room temperature. Secondary antibodies used were A9169 (goat antirabbit immunoglobulin G (IgG), 1:80,000; Sigma-Aldrich) and A9044 (rabbit antimouse IgG, 1:80,000; Sigma-Aldrich). Blots were washed twice in PBST, and bands were visualized using the ECL Plus Western Blotting detection system (GE Healthcare, Little Chalfont, Buckinghamshire, UK) and exposed to CL-XPosure Film (Thermo Scientific, Rockford, IL, USA). Immunoreactive bands were quantified using background subtraction with a Bio-Rad densitometer and Bio-Rad Multi-Analyst software (Bio-Rad, Hercules, CA, USA). with the following approximate molecular weights: 224 kDa (dimer) and 112 kDa (monomer) for mGluR5; 73 kDa for FMRP; 52 kDa for GABRβ3 (We have observed a range of molecular weights for GABRβ3 including 52 kDa and 56 kDa which agrees with the expected molecular weight of 51-56 kDa according to the antibody data sheet from Novus Biologicals, Inc. and from previously published results [29-31]); 50, 46, 42 and 38 kDa for GFAP (all bands measured together); 46 kDa for NSE; and 42 kDa for β-actin. The results obtained were based on at least two independent experiments.

### Statistical analysis

Statistical analysis of protein data was performed as previously described [26,27]. We investigated the potential confounding effects of age, postmortem interval and gender by examining group differences with postmortem intervals as covariates. We further analyzed data on the basis of stratification by age groups, that is, adults (older than 18 years of age) and children (ages 18 and younger). Effect sizes were calculated using Cohen's *d *statistic, with values >0.8 considered a large effect [32].

## Results

**Figure 1:**
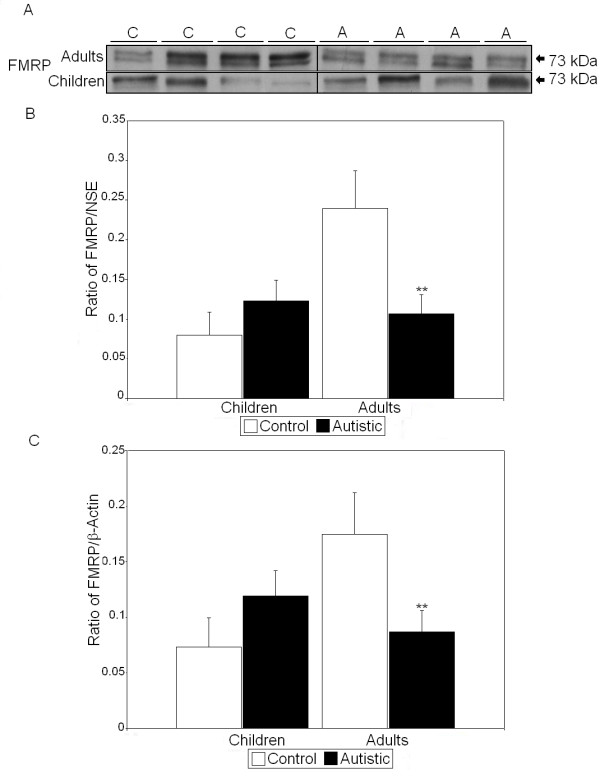
**Expression of FMRP in superior frontal cortex of people with autism and matched controls.(A)** Representative samples of fragile X mental retardation protein (FMRP) from controls (C) and people with autism (A). **(B) **Mean FMRP/neuron-specific enolase (NSE) ratios for people with autism (filled histogram bars) and controls are shown for Brodmann's area 9 (BA9). **(C) **Mean FMRP/β-actin ratios for people with autism (filled histogram bars) and controls are shown for BA9. Error bars express standard error of the mean. **P < 0.05.

**Figure 2:**
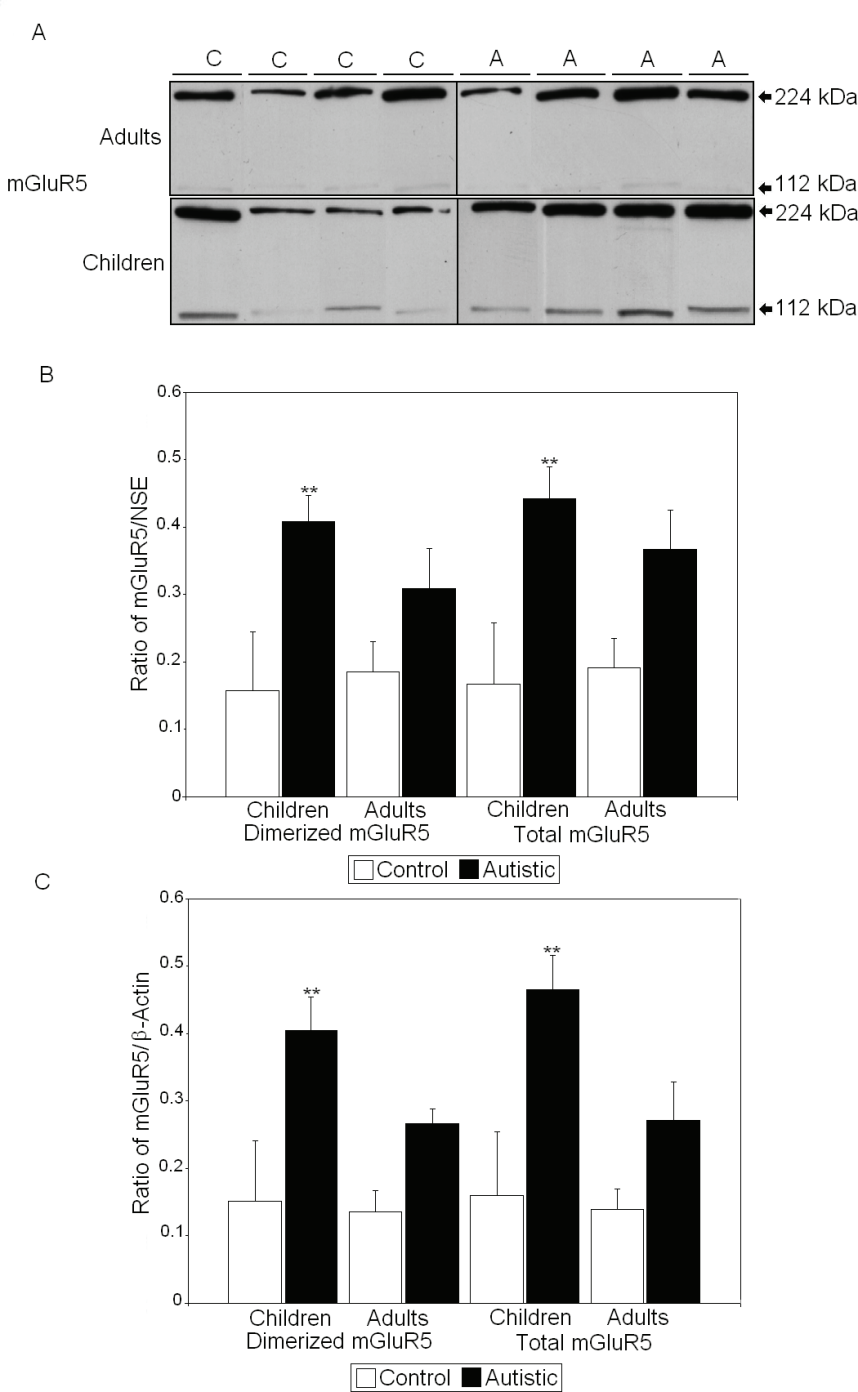
**Expression of mGluR5 in superior frontal cortex of people with autism and matched controls.(A) **Representative samples of metabotropic glutamate receptor 5 (mGluR5) from controls (C) and people with autism (A). **(B) **Mean mGluR5 dimer and monomer/neuron-specific enolase (NSE) ratios for people with autism (filled histogram bars) and controls are shown for Brodmann's area 9 (BA9). **(C) **Mean mGluR5/β-actin ratios for people with autism (filled histogram bars) and controls are shown for BA9. Error bars express standard error of the mean. **P < 0.05.

**Figure 3:**
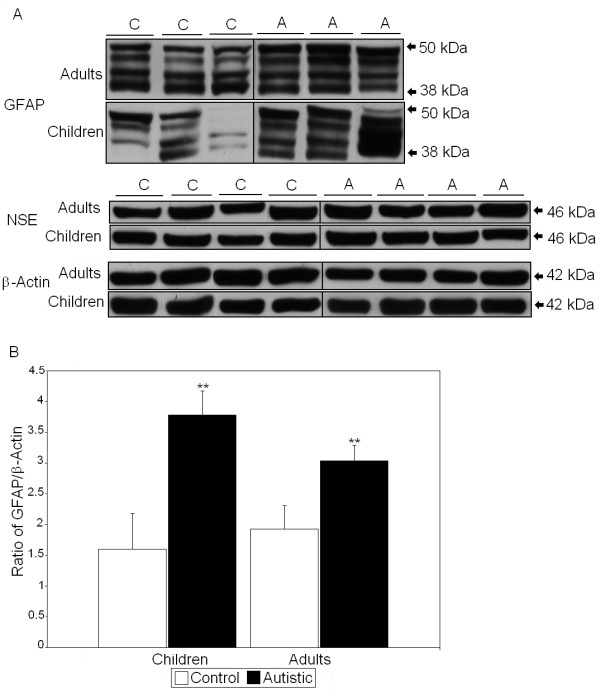
**(A) Representative samples of glial fibrillary acidic protein (GFAP), neuron-specific enolase (NSE) and β-actin from controls (C) and people with autism (A)**. **(B) **Mean GFAP/β-actin ratios for people with autism (filled histogram bars) and controls are shown for Brodmann's area 9 (BA9). Error bars express standard error of the mean. **P < 0.05.

**Figure 4:**
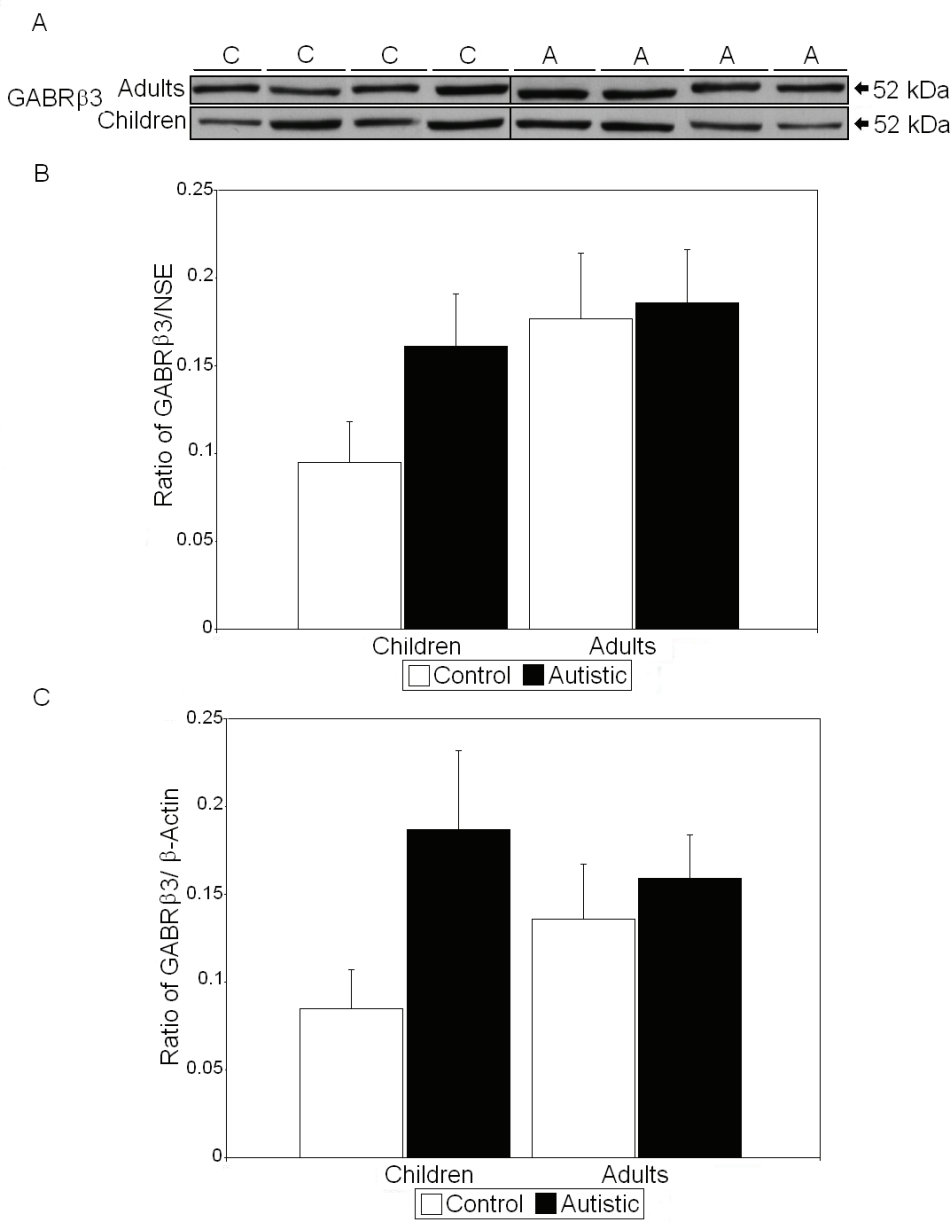
**Expression of GABRβ3 in superior frontal cortex of people with autism and matched controls. (A)** Representative samples of γ-aminobutyric acid (GABA) A receptor β3 (GABRβ3) from controls (C) and people with autism (A). **(B) **Mean GABRβ3/neuron-specific enolase (NSE) ratios for people with autism (filled histogram bars) and controls are shown for Brodmann's area 9 (BA9). **(C) **Mean GABRβ3/β-actin ratios for people with autism (filled histogram bars) and controls are shown for BA9. Error bars express standard error of the mean.

All values derived using Western blot analysis were normalized against two housekeeping proteins: NSE and β-actin. Previous studies of FMRP have shown that two to five distinct bands of FMRP may appear on SDS-PAGE and Western blots which are produced by alternative splicing of the *FMR1 *gene [33,34]. We observed up to two bands, which were measured together. We observed that there were significant reductions in the FMRP/NSE ratio (55%, *P *< 0.017) and the FMRP/β-actin ratio (50%, *P *< 0.042) in BA9 of adults with autism compared with controls (*P *< 0.026) (Figure 1 and Tables 2 and 3). In BA9 of children with autism, there were significant increases in the dimerized mGluR5/NSE ratio (159%, *P *< 0.013) and total mGluR5/NSE ratio (165%, *P *< 0.014) (Figure 2 and Table 2). Similarly, there were significant increases in both the dimerized mGluR5/β-actin ratio (168%, *P *< 0.023) and the total mGluR5/β-actin ratio (191%, *P *< 0.011) (Table 3). We previously observed a significant difference in dimerized mGluR5 vs. total mGluR5 in vermis of children with autism [25]; however, a similar result was not seen in BA9 (Table 2). GFAP was found to be significantly elevated in BA9 of both children (136%, *P *< 0.012) and adults (58%, *P *< 0.033) with autism (Figure 3 and Tables 2 and 3). We observed no significant differences in protein levels for NSE (Figure 3 and Table 2) or β-actin (Figure 3 and Table 3), suggesting that the observed changes were not due to changes in neuronal cell numbers between people with autism and matched controls. Finally, there was no significant change in GABRβ3 between people with autism and matched controls (Figure 4 and Tables 2 and 3), despite large effect sizes.

**Table 2:** Western blot analysis results for FMRP, mGluR5, GABRβ3 and NSE and their ratios in BA9^a^

Children	Control	Autistic	Change	*P *value	Cohen's *d*
FMRP/NSE	0.08 ± 0.057	0.123 ± 0.069	↑53%	ns	0.66
mGluR5 dimer/NSE	0.158 ± 0.175	0.409 ± 0.101	↑159%	0.013^b^	1.93^b^
mGluR5 total/NSE	0.167 ± 0.182	0.443 ± 0.123	↑165%	0.014^b^	1.90^b^
mGluR5 dimer/mGluR5 total	0.867 ± 0.154	0.93 ± 0.042	↑7.2%	ns	0.66
GABRβ3/NSE	0.095 ± 0.052	0.161 ± 0.086	↑69%	ns	0.86^b^
NSE	17.4 ± 0.642	19.20 ± 2.67	↑10.4%	ns	--

**Adults**	**Control**	**Autistic**	**Change**	***P *** **value**	**Cohen's *****d***

FMRP/NSE	0.24 ± 0.094	0.107 ± 0.068	↓55%	0.017^b^	1.74^b^
mGluR5 dimer/NSE	0.186 ± 0.098	0.309 ± 0.188	↑66%	ns	0.74
mGluR5 total/NSE	0.191 ± 0.099	0.367 ± 0.187	↑92%	ns	1.05^b^
mGluR5 dimer/mGluR5 total	0.974 ± 0.023	0.982 ± 0.011	↑0.8%	ns	0.50
GABRβ3/NSE	0.177 ± 0.082	0.186 ± 0.103	↑5.1%	ns	0.09
NSE	17.5 ± 3.17	18.8 ± 2.3	↑1.3%	ns	-

^a^FMRP, fragile X mental retardation protein; mGluR5, metabotropic glutamate receptor 5; GABRβ3, γ-aminobutyric acid (GABA) A receptor β3; NSE, neuron-specific enolase; BA9, Brodmann's area 9; ns, not significant; ^b^statistically significant.

**Table 3:** Western blot analysis results for FMRP, mGluR5, GABRβ3, GFAP and β-actin and their ratios in BA9^a^

Children	Control	Autistic	Change	*P *value	Cohen's *d*
FMRP/β-actin	0.073 ± 0.054	0.119 ± 0.062	↑63%	ns	0.76
mGluR5 dimer/β-actin	0.151 ± 0.179	0.405 ± 0.131	↑168%	0.023^b^	1.71^b^
mGluR5 total/β-actin	0.16 ± 0.187	0.466 ± 0.131	↑191%	0.011^b^	2.01^b^
GABRβ3/β-actin	0.085 ± 0.049	0.187 ± 0.128	↑120%	ns	0.96^b^
GFAP/β-actin	1.6 ± 1.16	3.78 ± 0.962	↑136%	0.012^b^	2.09^b^
β-actin	20.1 ± 2.94	18.3 ± 4.79	↓8.9%	ns	-

**Adults**	**Control**	**Autistic**	**Change**	***P *** **value**	**Cohen's *****d***

FMRP/β-actin	0.175 ± 0.075	0.087 ± 0.055	↓50%	0.042^b^	1.43^b^
mGluR5 dimer/β-actin	0.136 ± 0.069	0.267 ± 0.175	↑96%	ns	0.87^b^
mGluR5 total/β-actin	0.139 ± 0.069	0.272 ± 0.178	↑96%	ns	0.87^b^
GABRβ3/β-actin	0.136 ± 0.069	0.159 ± 0.087	↑17%	ns	0.27
GFAP/β-actin	1.93 ± 0.762	3.04 ± 0.758	↑58%	0.033^b^	1.46^b^
β-actin	23.2 ± 1.97	21.7 ± 3.28	↓6.5%	ns	-

^a^FMRP, fragile X mental retardation protein; mGluR5, metabotropic glutamate receptor 5; GABRβ3, γ-aminobutyric acid (GABA) A receptor β3; GFAP, glial fibrillary acidic protein; BA9, Brodmann's area 9; ns, not significant. ^b^statistically significant.

There were no significant differences between individuals with autism and controls related to age or postmortem interval (PMI) (Tables 2 and 3). We examined the confounding effects of age and PMI on our results and found no impact of age on the levels of any proteins (data not shown). There was an impact of PMI on the FMRP/β-actin ratio. However, as there was no significant difference in PMI between the two groups, the impact of PMI on the FMRP/β-actin ratio was not meaningful.

The effect of gender was examined in both children and adults. For children, there were no girls in the control group. However, when control boys were compared with boys with autism, the results for FMRP and GABRβ3 remained nonsignificant and total mGluR5 and GFAP remained significantly elevated, while the dimerized mGluR5/β-actin ratio lost significance (data not shown). In adults, it was impossible to evaluate the effect of gender on levels of FMRP and GFAP owing to low power, and mGluR5 and GABRβ3 remained nonsignificant (data not shown).

Mental retardation, seizures and use of anticonvulsants were all confounded within the autism group. Neither seizure disorder nor anticonvulsant use affected the Western blot analysis results (data not shown). In comparing people with autism who had seizure disorder with people with autism without seizure disorder, we found that there were no significant differences for FMRP in children, while this comparison could not be tested in adults because of the low sample size. However, there was a 47% reduction in FMRP in adults with autism and seizure disorder compared with adults with autism without seizure disorder (data not shown). In only one instance was mental retardation a significant predictor of differences in Western blot analysis results, and that was for the GFAP/β-actin ratio in adults. When that individual (subject 6469) was removed from the analysis, the difference remained significant between people with autism and controls.

Effect sizes were calculated for the comparisons using Cohen's *d *statistic where an effect size >0.8 is considered a large effect [32]. In children, the comparisons of mGluR5 dimer/NSE ratio, mGluR5 total/NSE ratio, GABRβ3/NSE ratio, mGluR5 dimer/β-actin ratio, mGluR5 total/β-actin ratio, GABRβ3/β-actin ratio and GFAP/β-actin ratio displayed large effect sizes (Tables 2 and 3). In adults, the comparisons of FMRP/NSE ratio, mGluR5 total/NSE ratio, FMRP/β-actin ratio, mGluR5 dimer/β-actin ratio, mGluR5 total/β-actin ratio and GFAP/β-actin ratio between the two groups consisted of large or very large effect sizes (Tables 2 and 3).

## Discussion

In the current study, we found reduced protein expression of FMRP in BA9 of adults with autism, increased expression of mGluR5 in children with autism, increased expression of GFAP in both children and adults with autism and no significant difference in GABRβ3 expression. With the exception of no change in GABRβ3 expression, these changes in the prefrontal cortex (PFC; that is, BA9) mirror what we have previously observed in cerebellar vermis of people with autism [25].

We are the first to demonstrate significant changes in protein for FMRP in BA9 of adults with autism. Recent imaging studies have shown reduced activation of the PFC during cognitive performance tasks in individuals with FXS [35-37]. Hoeft *et al*. [36] found reduced activation in the right ventrolateral PFC in adolescent boys with FXS during a go vs. no go executive function task. Similarly, in a study of females with FXS, there was reduced activation of the PFC and striatum during a working memory task [35]. Reduced activation of the PFC has also been shown in females with FXS during mathematical processing [37]. Taken together, these studies indicate impairment of multiple PFC-mediated functions in people with FXS. The reduction in FMRP in BA9 in adults with autism may result in similar changes in brain activation and cognitive processing in individuals with autism.

Dopamine plays a critical role in cognitive functions in the PFC [38,39], and recent studies involving *Fmr1*-knockout mice have shown a role of FMRP in dopamine modulation in the PFC [40,41]. Such a role for FMRP may help to explain learning and memory deficits in people with FXS [40,41]. Our finding of reduced FMRP expression in BA9 of adults may help to explain cognitive deficits in people with autism.

In contrast to our studies of vermis, we did not observe a significant reduction in GABRβ3 in BA9 of adults with autism. However, previous experiments in our laboratory using a different set of BA9 tissue from controls and individuals with autism also failed to show any significant difference in protein expression of GABRβ3 (SHF and TJ Reutiman, unpublished data). These results may reflect regional differences in GABRβ3 protein expression in the brains of people with autism.

Our finding regarding GFAP confirms our earlier findings of significant elevation of GFAP in BA9 of a different set of people with autism [42]. This increased level of GFAP is consistent with previous findings of astroglial activation in the brains of individuals with autism [43-45], suggesting dysregulation of the immune system. Coexpression of GFAP and FMRP has been demonstrated by immunocytochemical studies in the hippocampi of embryonic and postnatal mice, but not in adults [46,47]. Significantly increased GFAP has been observed in the cerebellum [48] and in the striatum, hippocampus, and cerebral cortex [49] of fragile X-knockout mice. Ellegood *et al*. [48] suggested that the loss of FMRP and increase in cerebellar GFAP could contribute to anatomical changes in the cerebellum.

The significant elevation of mGluR5 protein in children with autism is an interesting finding in light of the connection between group 1 mGluRs and FMRP in FXS, which are believed to act in opposition to one another. It has been proposed that in the absence of FMRP, unchecked mGluR-dependent protein synthesis leads to the pathology of FXS [50,51]. A number of recent findings in *Fmr1*-knockout mice have shown that the use of mGluR5 inhibitors, such as 2-methyl-6-(phenylethynyl)-pyridine (MPEP), rescues FXS phenotypes [52,53]. Recent experiments by Silverman *et al*. [54,55] have shown that MPEP was useful in the treatment of repetitive grooming behavior in the BTBR mouse model of autism by antagonizing mGluR5 receptors. This new model adds to the previous animal models for autism and FXS and helps to expand our understanding of the role of mGluR5 dysfunction.

The increased expression of mGluR5 in children with autism suggests pathologic activation of mGluR5 which may be prenatal or early postnatal. This activation may lead to seizures, cognitive problems and perhaps brain morphological abnormalities in children with autism and FXS. While FMRP levels are similar in children with autism and controls, overactive mGluR5 signaling may be strong enough to overcome FMRP's inhibitory effect. In adults, there continue to be elevated levels of dimerized mGluR5 (66% increase for mGluR5/NSE ratio and 92% increase for mGluR5/β-actin ratio) and total mGluR5 (96% increase for both mGluR5/NSE and mGluR5/β-actin ratios), although they do not reach the level of statistical significance. At the same time, adults with autism have significantly lower levels of FMRP. This reduction in FMRP reduces its inhibitory effect on mGluR5 signaling, resulting in a continuation of the phenotypes observed in children with autism and FXS.

The interaction between FMRP and mGluR5 also presents a potential means of treatment using allosteric modulators of mGluR5. In a pilot study, treatment of patients with FXS with lithium, which reduces mGluR-activated translation, led to improved scores on the Aberrant Behavior Checklist-Community Edition and was well-tolerated [56]. Additionally, fenobam, an anxiolytic drug that acts as an inhibitor of mGluR5 [57], has been shown to correct prepulse inhibition deficits in people with with FXS in a recent open-label pilot study [58]. Norbin is a positive modulator of endogenous mGluR5 that enhances mGluR5 signaling [59]. Reduction in norbin has been shown to lead to a consequent reduction in mGluR5 expression, and norbin-knockout mice show enhanced locomotor activity similar to that of mice treated with MPEP [59]. It may be the case that allosteric modulators of mGluR5 or targeting of naturally occurring modulators of mGluR5 could have similar effects on people with autism and warrant future exploration. A further avenue of treatment may be the use agents which raise the levels of GABA in the brains of individuals with autism. Recently, Heulens *et al*. [60] reported on ganaxolone as a potential GABA_A _receptor agonist in the treatment of people with FXS. Certainly this agent, as well as GABA_B _receptor agonists such as arbaclofen, could be investigated in the treatment of autism.

## Conclusions

We observed reduced expression of FMRP in BA9 of adults with autism, and increased expression of mGluR5 in children with autism, compared with matched controls. These findings mirror results observed in our laboratory in cerebellar vermis of people with autism. These observed changes in protein expression of FMRP in adults and mGluR5 in children are likely to contribute to cognitive deficits and the presence of comorbid seizure disorder in individuals with autism. The interaction between FMRP and mGluR5 provides a potential avenue for treatment through the use of allosteric modulators.

## Competing interests

The authors declare that they have no competing interests.

## Authors' contributions

SHF conceived of the study, participated in its design and contributed to the drafting of the manuscript. TDF performed the Western blot analysis, was involved in statistical analyses and contributed to the drafting of the manuscript. Both authors read and approved the final manuscript.
